# Construction and analysis of the abnormal lncRNA–miRNA–mRNA network in hypoxic pulmonary hypertension

**DOI:** 10.1042/BSR20210021

**Published:** 2021-08-26

**Authors:** Jie Liu, Yishu Deng, Zeqin Fan, Shuanglan Xu, Li Wei, Xiaoxian Huang, Xiqian Xing, Jiao Yang

**Affiliations:** 1 Department of Respiratory Medicine, The Fourth Affiliated Hospital of Kunming Medical University, Kunming 650021, Yunnan, China; 2 Department of Respiratory Medicine, The Affiliated Hospital of Yunnan University, The Second People’s Hospital of Yunnan Province, Kunming 650021, Yunnan, China; 3 First Department of Respiratory Medicine, The First Affiliated Hospital of Kunming Medical University, Kunming 650032, Yunnan, China

**Keywords:** competing endogenous RNA, differently expressed genes, hypoxic pulmonary hypertension, long non-coding RNAs, miRNA, mRNA

## Abstract

The incidence of hypoxic pulmonary hypertension (HPH) is increasing. Accumulating evidence suggests that long noncoding RNAs (lncRNAs) play an important role in HPH, but the functions and mechanism have yet to be fully elucidated. In the present study, we established a HPH rat model with 8 h of hypoxia exposure (10% O_2_) per day for 21 days. High-throughput sequencing identified 60 differentially expressed (DE) lncRNAs, 20 DE miRNAs and 695 DE mRNAs in rat lung tissue. qRT-PCR verified the accuracy of the results. The DE mRNAs were significantly enriched in immune response, inflammatory response, leukocyte migration, cell cycle, cellular response to interleukin-1, IL-17 signalling pathway, cytokine–cytokine receptor interaction and Toll-like receptor signalling pathway. According to the theory of competing endogenous RNA (ceRNA) networks, lncRNA–miRNA–mRNA network was constructed by Cytoscape software, 16 miRNAs and 144 mRNAs. The results suggested that seven DE lncRNAs (Ly6l, AABR07038849.2, AABR07069008.2, AABR07064873.1, AABR07001382.1, AABR07068161.1 and AABR07060341.2) may serve as molecular sponges of the corresponding miRNAs and play a major role in HPH.

## Introduction

Hypoxic pulmonary hypertension (HPH) is generally characterized as atypical contraction and remodelling of the small pulmonary vessels caused by abnormal activation of the local pulmonary vasoconstriction system and proliferation of pulmonary smooth muscle cells [1]. HPH is common in almost all advanced stages of chronic lung disease, and the prevalence of HPH has increased in both adults and children in recent years [2]. Although many studies have investigated HPH, its pathogenesis has not been fully elucidated and must be explored from a new perspective.

Long noncoding RNAs (lncRNAs), which are more than 200 nucleotides in length, were once considered unrelated transcriptional waste with no biological function [3]; however, the competing endogenous RNA (ceRNA) hypothesis indicates that mRNA, transcriptional pseudogenes and lncRNAs interact with each other by competing for microRNA (miRNA) binding [4]. LncRNAs, including ceRNAs, have been found to be widely involved in the biological processes of many diseases by regulating the amount and function of miRNAs, thus affecting downstream signalling pathways [5,6]. In addition, many lncRNAs have been shown to be differentially expressed (DE) in HPH rats compared with healthy rats [7]. Subsequently, lncRNA-MEG3 and lncRNA-Tug1 have been shown to contribute to HPH as ceRNAs [8,9]. However, no studies have comprehensively analysed the correlation among lncRNAs, miRNAs and mRNAs in HPH. Thus, construction of lncRNA–miRNA–mRNA ceRNA networks is necessary. We screened for DE RNAs through high-throughput sequencing and constructed lncRNA–miRNA–mRNA ceRNA networks. Our study provides a novel theoretical basis for the pathogenesis of HPH from the perspective of ceRNA.

## Materials and methods

### Animal model

Twelve male specific pathogen-free Sprague-Dawley rats aged 6-8 weeks (150–200 g) were purchased from the Animal Centre of Kunming Medical University. The animal experiments were carried out in the Experimental Animal Center of Kunming Medical University and approved by the Ethics Committee of Kunming Medical University (KMMU2020207).

After a 3-day adaptation period, rats were randomly divided into the HPH group and the healthy group. According to previously reported methods [10], rats in the HPH group were exposed to normal pressure hypoxia (9.5–10.5% O_2_) 8 h a day for 21 days to construct the HPH rat model. Rats in the healthy group were housed under normoxic conditions.

### RVSP, RVHI and WA (%) measurements

After 3 weeks in hypoxic or normoxic environments, rats were injected with sodium pentobarbital. When the anaesthetized rats had a decreased respiratory rate, complete muscle relaxation, disappearance of the eyelid reflex and a weak corneal reflex, the experiment was started. A polyethylene catheter was inserted from the right jugular vein to the right ventricle, then the right ventricle systolic pressure (RVSP) was recorded using the BL-420F biometric function test system. After the rats were killed by exsanguination under surgical levels of anaesthesia, lung and heart tissues were separated and collected. The right lung tissue was divided into two parts as follows: one part was used for sequencing, and one part was stored at −80°C. The left lung was saturated with 4% formaldehyde. Atrial tissue was removed from the collected heart, and the right ventricle, left ventricle and ventricular septum were separated. The index of right ventricular hypertrophy (RVHI) was measured using the following equation: RVHI = right ventricle / (left ventricle + ventricular septum). After fixation in 4% formaldehyde for 2 weeks, lung tissues were dehydrated with different concentrations xylene and then embedded with paraffin. Lung sections were stained with hematoxylin and eosin solutions, and then photographed using a light microscope. The ratio of wall area (WA%) of the pulmonary arterioles (approximate diameter of 100 µm and concomitant with terminal bronchiole) was calculated using the following formula to reflect the remodelling of the pulmonary microvasculature: WA% = (1 – lumen area / vessel total area).

### Sequencing and identification of DE lncRNAs and DE mRNAs

Three right lung tissues from each of the two groups were randomly selected for sequencing. Total RNA was extracted from the lung tissues using TRIzol Reagent (Ambion Co., Carlsbad, U.S.A.), and the RNA concentration and purity were determined by oligosaccharide electrophoresis and an Agilent 2100 Bioanalyzer. The rRNA in the total RNA was removed using a ribosomal RNA removal kit, and 200–300 bp RNA fragments were generated by ion interruption. The first strand of cDNA was synthesized using 6-base random primers and reverse transcriptase, and the second strand of cDNA was synthesized using the first strand of cDNA as the template. After library construction, PCR amplification was used for library fragment enrichment, and the library selection was then performed according to the fragment size with a library size ranging from 300 to 400 bp. An Agilent 2100 Bioanalyser was used to conduct quality inspection of the library, and paired-end sequencing was then performed based on an Illumina sequencing platform. The Ensembl database (http://www.ensembl.org/) was selected as the reference genome.

The raw data were filtered, and the resulting clean data were aligned to the rat reference genome. We also used CPC, CNCI and Pfamscan software to determine whether these new transcripts that are formed by resplicing of exons based on sequencing principles and are different from known RNAs that have the protein-coding ability to obtain highly reliable lncRNAs. We considered that the new transcripts with no coding potential determined by all three software programs were highly reliable lncRNAs for subsequent analysis. HTSeq statistics were used to obtain the read count value for each gene, and fragments per kilobase of transcript per million mapped reads (FPKM) was used to standardize lncRNA and mRNA expression. EdgeR [11], a software package of R for the comprehensive analysis of count data, was used to identify differentially expressed (DE) lncRNAs and DE mRNAs by a threshold *P*-value <0.05 and log_2_ fold change (FC) >1 or <1.

### Sequencing and identification of DE miRNA

Total RNA was extracted from the randomly selected lung tissues using TRIzol Reagent (Ambion Co., Carlsbad, U.S.A.). The TruSeq Small RNA Sample Prep Kit (Illumina) was used to construct the small RNA library after detection of total RNA concentration and purity. After PCR amplification and addition of the sequencing joint and index, gel electrophoresis was used to purify the library. An Agilent 2100 Bioanalyzer and Quant-iT PicoGreen dsDNA Assay Kit were used for quality detection and quantitative analysis of the library, respectively. Finally, the optimal sample size was selected and sequenced using an Illumina sequencing platform.

The raw data were processed to remove joints and quality filtering, and duplicated sequences were removed to obtain clean data. The clean data were mapped in the miRbase database to identify known miRNAs. According to the sequence number of miRNA maturation, the miRNA read count value was statistically measured. Counts per million (CPM) values were used to investigate the gene expression patterns of the samples as a whole. DE miRNAs were identified by using edgeR with a threshold *P*-value <0.05 and an absolute value of log_2_ (FC) <1.

### Functional enrichment analysis

Metascape (http://metascape.org/) [12] was used to perform Gene Ontology (GO) and Kyoto Encyclopaedia of Genes and Genomes (KEGG) pathway analysis for DE mRNAs. Biological processes (BP), cellular components (CC) and molecular functions (MF) in GO analysis were performed. The GO and KEGG analysis results were considered statistically significant with a threshold *P*-value <0.05. The results were saved as a text file, and ggplot2 packages in R were used for visual analysis.

### Protein–protein interaction network

Protein–protein interaction (PPI) networks of DE mRNAs were constructed by STRING (https://string-db.org/). The minimum required interaction score was 0.40, and the disconnected nodes in the network were removed.

### Construction of the ceRNA regulatory network

According to the regulation theory of competing endogenous RNA (ceRNA) networks, lncRNAs, including miRNA sponges, compete with mRNA through miRNA response elements (MREs) [4]. Therefore, in a ceRNA regulatory network, lncRNAs and mRNAs should show the same change trends, while miRNAs should show an opposing change. The MiRanda database was used to obtain the relationship between DE lncRNAs and DE miRNAs as well as between DE miRNAs and DE mRNAs. To analyse the interaction between lncRNAs and miRNAs, DE lncRNAs were annotated through the Ensemble database. Only known DE lncRNAs were included in the ceRNA network. The DE lncRNA–DE miRNA interactions and DE miRNA–DE mRNA interactions were then combined into the DE lncRNA–DE miRNA–DE mRNA network. Cytoscape software (version 3.7.0) was used to build this ceRNA network.

### Quantitative polymerase chain reaction (qPCR) validation

The ceRNAs with large differential expression in the lncRNA-miRNA-mRNA network were selected for validation by qPCR. Primers were designed using the NCBI Primer-BLAST website (https://www.ncbi.nlm.nih.gov/tools/primer-blast/), and the sequences are shown in Table 1. U6 and GAPDH were used as internal references for noncoding RNA and mRNA, respectively. Total RNA from the lung tissue was extracted using TRIzol (Ambion Co., Carlsbad, U.S.A.). Complementary deoxyribose nucleic acid was synthetized using a reverse transcription kit (TIANGEN Biotech Co., Ltd., Beijing, China). Quantitative PCR was performed using an ABI Prism 7300HT Real Time PCR amplification apparatus. The results were analysed with the 2^−ΔΔCt^ method.

**Table 1:** Primers for quantitative real-time PCR (qRT-PCR)

Gene names	Category	Primer sequence (5′-3′)
Ly61	lncRNA	Forward-GGCCTCTTCTGCATCCTGTT
		Reverse-CCTGGGTCCTAAGCAAAGGG
AABR07068161.1	lncRNA	Forward-ATGAGCAGATTACGGGCAGG
		Reverse-CTGGATGAGATGGAGACCGC
AABR07001382.1	lncRNA	Forward-GAGCCATCCATGAACTCGGT
		Reverse-TCATTGCCTTTCCTATGCTGC
miR-21-5p	miRNA	Forward-TAGCTTATCAGACTGATGTTGA
		Universal downstream primer of kit
miR-485-5p	miRNA	Forward-GAGGCTGGCCGTGATGAATTC
		Universal downstream primer of kit
Cxcl11	mRNA	Forward-CGCGGAGTGAAAGTGGTCAA
		Reverse-AGCCTTCAGGGTAACAATCACT
Hoxa1	mRNA	Forward-CAGGAAGCAGACCCACCAAG
		Reverse-GACCCACGTAGCCATACTCTC
Trim29	mRNA	Forward-CTGAAAGGCTATCCCTCCCTC
		Reverse-TGGCCGGTAGTGAGACAGTA
Myadml2	mRNA	Forward-TGTTGCCGCGGAAGATAGAG
		Reverse-CAGCGCCTAAGTGCAGGTAT

### Statistical analysis

All data are presented as the mean ± standard deviation. An independent sample *t*-test was used to compare data between different groups with SPSS17.0 software. *P*<0.05 was considered statistically significant.

## Results

### Successful construction of animal models

**Figure 1:**
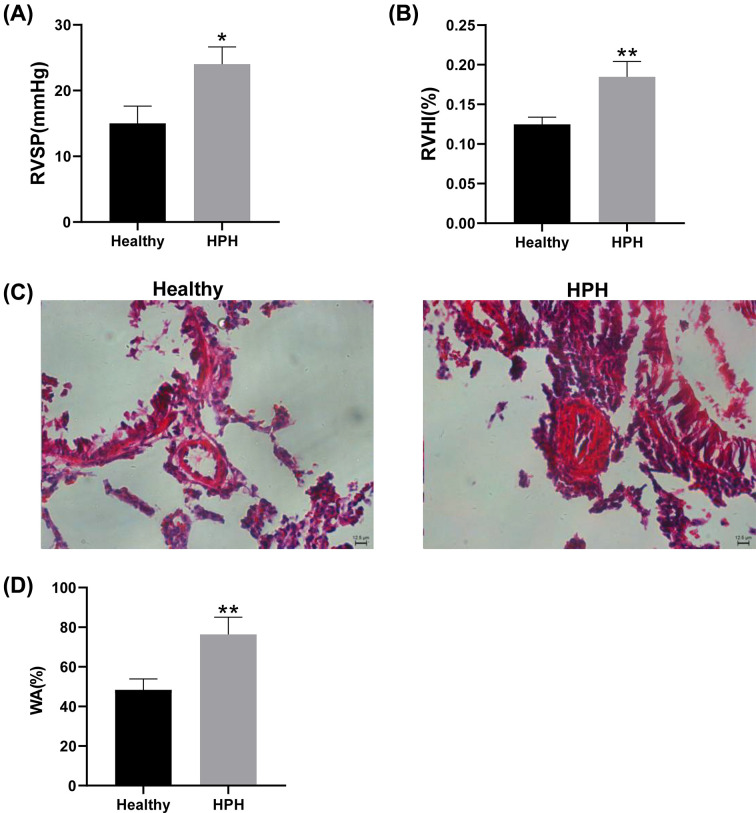
Successful construction of the HPH rat model (**A**) Right ventricular pressure (RVSP) in HPH and healthy rats. (**B**) Right ventricular hypertrophy (RVHI) was measured using the following formula: RVHI = right ventricle / (left ventricle + ventricular septum). (**C**) Haematoxylin and eosin (HE) staining of pulmonary arterial sections. (**D**) Wall area (WA) was calculated using the following equation: WA = 1 – lumen area / vessel total area; *P<0.05 and **P<0.01 versus healthy group.

In the six rats randomly selected for RNA sequencing, the rats in the HPH group showed significantly increased RVSP and RVHI compared with the healthy group (15.00 ± 2.65 mmHg versus 24.00 ± 2.65 mmHg and 0.12 ± 0.01 versus 0.18 ± 0.02, respectively; Figure 1A,B). At the same time, the wall of small pulmonary vessels in the HPH group was significantly thickened compared to that in the healthy group (Figure 1C). We next measured WA to quantitatively describe the changes in pulmonary small vessel remodelling. The results suggested that WA values were higher in the hypoxic group (Figure 1D). Together, these results demonstrated that the HPH rat model was successfully constructed.

### Identification of DE mRNAs, DE miRNAs and DE lncRNAs

**Figure 2:**
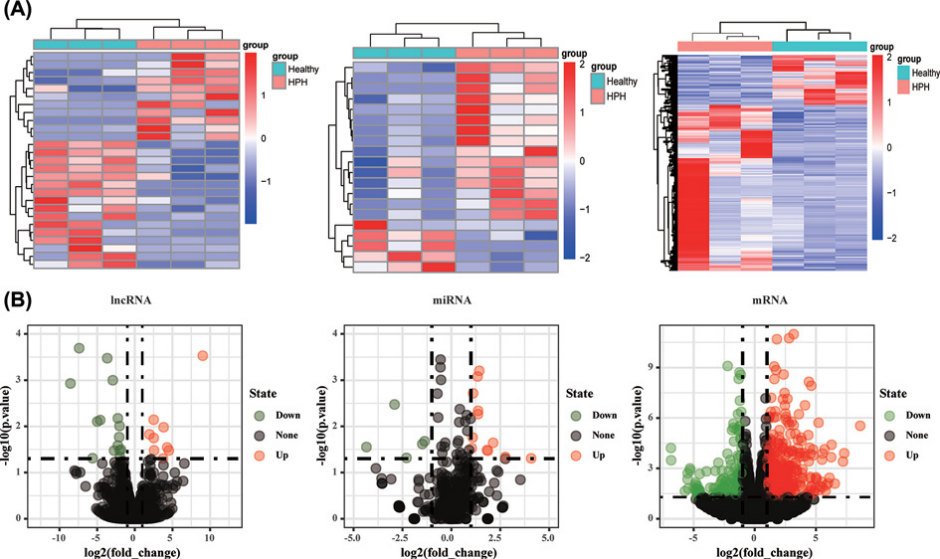
Identification of the differentially expressed (DE) genes in the HPH rat model (**A**) Heatmaps for the DE lncRNAs, DE miRNAs and DE mRNAs. Up-regulated genes are shown in red, and down-regulated genes are shown in green. (B) Volcano plots of the DE lncRNAs, DE miRNAs and DE mRNAs. The red, blue and black dots represent genes that are up-regulated, down-regulated and not significantly differentially expressed, respectively.

We obtained expression profile data of mRNAs, miRNAs and lncRNAs through high-throughput sequencing, and all data were uploaded to GSE159668. Through comparison to the healthy group, 60 DE lncRNAs (31 up-regulated and 29 down-regulated), 20 DE miRNAs (15 up-regulated and 5 down-regulated) and 695 DE mRNAs (534 up-regulated and 161 down-regulated) were screened with the threshold *P*-value <0.05 and the absolute value of log_2_ (FC) <1 (Supplementary Table S1). The heatmap and volcano map of noncoding RNAs (ncRNAs) and mRNAs are shown in Figure 2A,B.

### Functional analysis of DE lncRNAs, DE miRNAs and DE mRNAs

**Figure 3:**
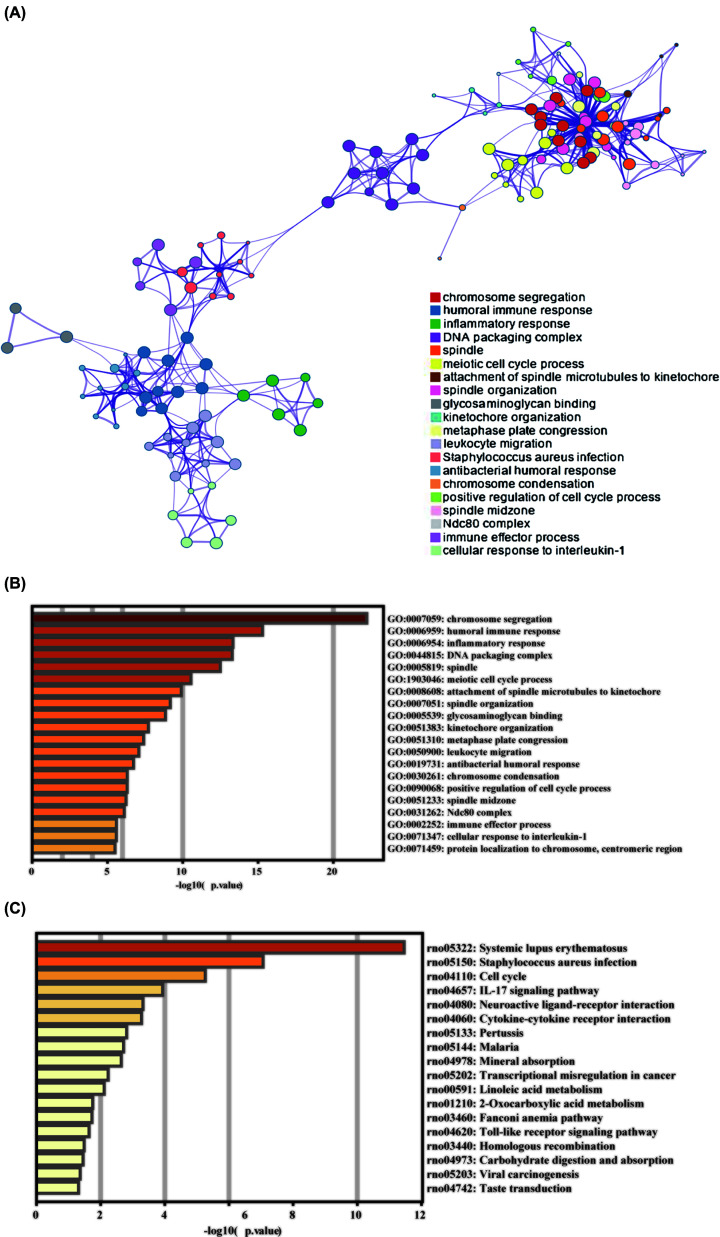
Functional enrichment of DE mRNAs (**A**) Top 20 clusters and their representative enriched terms (coloured by cluster ID). (**B**) Top 20 enriched terms on GO analysis. (**C**) Top 20 enriched terms on KEGG pathway analysis; DE, differentially expressed; GO, Gene Ontology; KEGG, Kyoto Encyclopaedia of Genes and Genomes.

Of the 695 DE mRNAs, many genes have been previously shown to play a role in different types of pulmonary hypertension. For example, MMP7 may participate in vascular remodelling in pulmonary hypertension [13]. Although there are weak associations between serum CXCL13 and disease severity, CXCL13 is overexpressed in both idiopathic pulmonary artery hypertension and chronic thromboembolic pulmonary hypertension [14]. Among the 20 DE miRNAs, miR-210-3p [15] and miR-21-5p [16] were associated with pulmonary hypertension. Of the 60 DE lncRNAs, only 16 DE lncRNAs were annotated, but their functions remain unclear. Metascape was used to perform functional enrichment of the DE mRNAs (Supplementary Table S2 and Figure 3A). GO analysis showed enrichment in chromosome segregation, humoural immune response, inflammatory response, leukocyte migration, cell cycle and cellular response to interleukin-1 (Figure 3B). In addition, KEGG pathway analysis revealed enrichment in the cell cycle, IL-17 signalling pathway, cytokine–cytokine receptor interaction and Toll-like receptor signalling pathway (Figure 3C).

### Construction of the lncRNA–miRNA–mRNA ceRNA network

**Figure 4:**
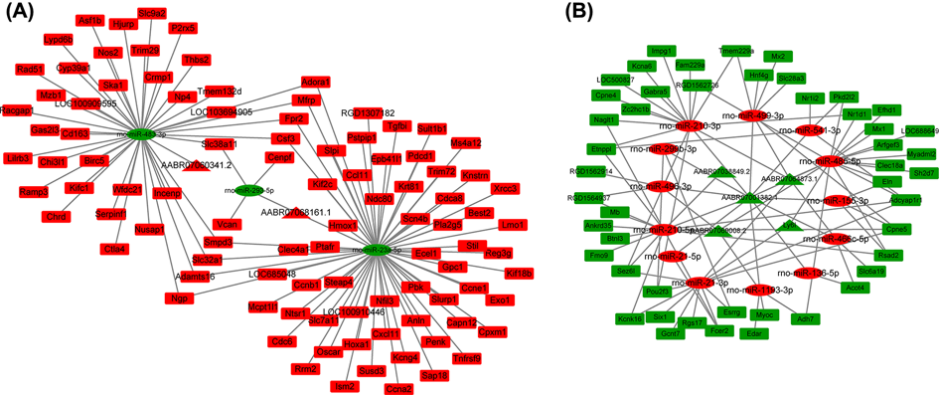
Construction of the lncRNA–miRNA–mRNA ceRNA regulatory network (**A**) The up-regulated lncRNA-down-regulated miRNA-up-regulated mRNA ceRNA regulatory network included 2 lncRNAs, 3 miRNAs and 97 mRNAs. (**B**) The down-regulated lncRNA-up-regulated miRNA-down-regulated mRNA ceRNA regulatory network included 5 lncRNAs, 13 miRNAs and 47 mRNAs. Red nodes indicate up-regulated RNAs, and green nodes indicate down-regulated RNAs. Triangles, ovals and rectangles indicate DE lncRNAs, DE miRNAs and DE mRNAs, respectively; ceRNA, competing endogenous RNA; DE, differentially expressed; lncRNAs, long noncoding RNAs; miRNAs, microRNAs.

The MiRanda dataset (http://www.microrna.org/microrna/) was used to predict the targets of DE ncRNAs among the DE RNAs. The results indicated that two up-regulated lncRNAs were predicted to have three down-regulated DE miRNAs, and the three down-regulated miRNAs were predicted to target 97 up-regulated DE mRNAs. Combining the lncRNA–miRNA pairs and miRNA–mRNA pairs, the ceRNAs of up-regulated lncRNAs–down-regulated miRNAs–up-regulated mRNAs were constructed in Cytoscape software (version 3.7.0) (Figure 4A). Using the same method, another ceRNA of down-regulated lncRNAs–up-regulated miRNAs–down-regulated mRNAs (including 5 lncRNAs, 13 miRNAs and 47 mRNAs, Figure 4B) was constructed.

### Functional analysis and PPI network of DE mRNAs in the lncRNA–miRNA–mRNA ceRNA network

**Figure 5:**
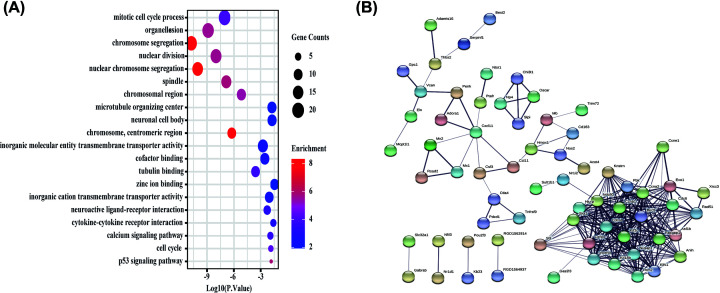
Functional enrichment and PPI networks of DE mRNAs in the ceRNA networks (**A**) Top 5 enriched terms in BP, CC, MF and KEGG analyses. (**B**) The PPI network of DE mRNAs in the ceRNA networks; BP, biological processes; CC, cellular components; MF, molecular functions; KEGG, Kyoto Encyclopedia of Genes and Genomes.

The 144 DE mRNAs in the ceRNA network were associated with the cell cycle, inorganic molecular entity transmembrane transporter activity, small molecule binding, cytokine–cytokine receptor interaction and p53 signalling pathway (Figure 5A). In addition, 19 hub genes were identified in the PPI network with MCODE score >15 (Figure 5B) and included Nusap1, Cenpf, Pbk, Ndc80, Kif2c, Ccnb1, Rrm2, Birc5, Cdca8, Ccna2, Cdc6, Racgap1, Kif18b, Exo1, Ska1, Kifc1, Hjurp, Incenp and Asf1b.

### qPCR validation of the ceRNA network genes

**Figure 6:**
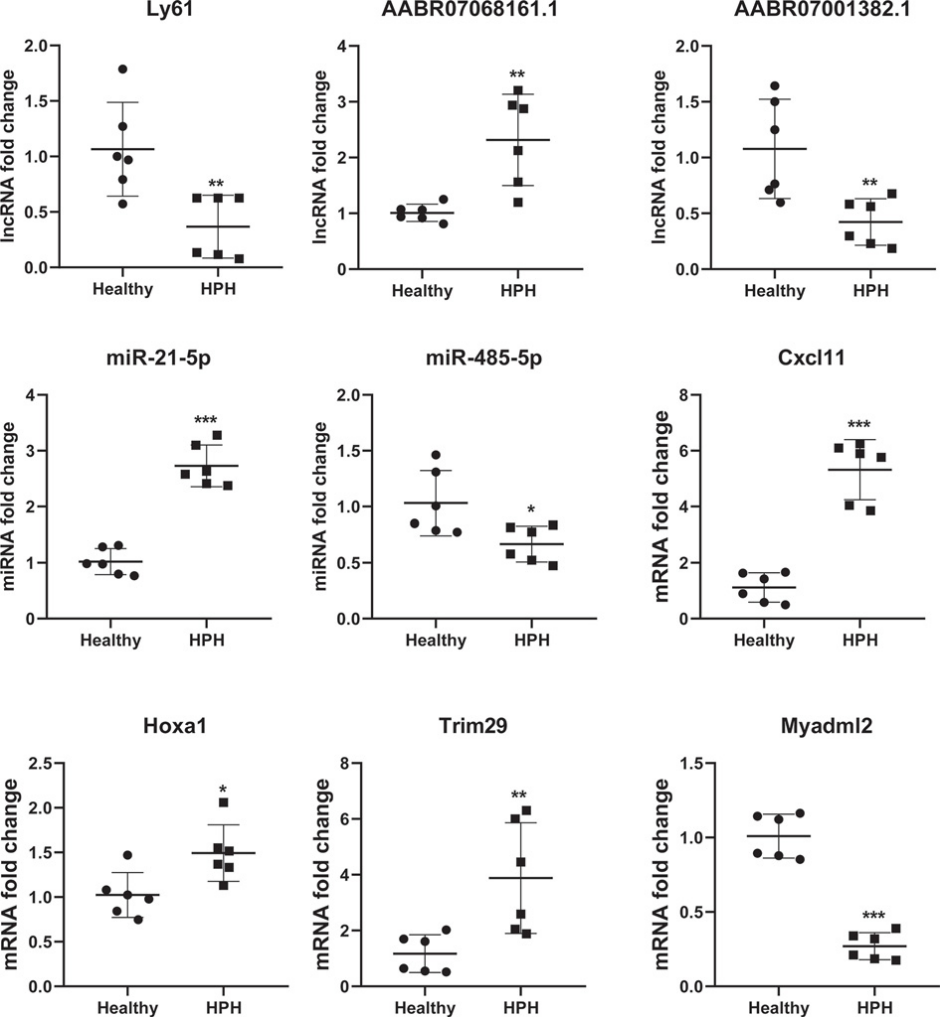
RNA expression in the HPH group compared with the healthy group *P<0.05, **P<0.01 and ***P<0.001 versus healthy group.

Three lncRNAs (Ly61, AABR07001382.1 and AABR07068161.1), two miRNAs (miR-21-5p and miR-485-5p) and four mRNAs (Cxcl11, Hoxa1, Trim29 and Myadml2) were selected for validation by qPCR to confirm the sequencing results. The expression of the nine selected RNAs was consistent with the sequencing results (Figure 6). Ly61, AABR07001382.1, miR-485-5p and Myadml2 showed down-regulated expression in the sequencing results, which was confirmed by qPCR. The expression of AABR07068161.1, miR-21-5p, Cxcl11, Hoxa1 and Trim29 in HPH rats was higher in both the high-throughput sequencing results and qPCR validation results.

## Discussion

HPH occurs frequently in patients with chronic obstructive pulmonary disease or idiopathic pulmonary fibrosis and in people living at high altitudes [17], and its incidence is increasing. Some early changes in HPH are difficult to detect with traditional research tools and expertise. Much effort has been afforded to finding new therapeutic targets and biomarkers for HPH [18–21]. Most studies, however, have focused only on mRNAs [18,20,21]. In recent years, lncRNAs have attracted increasing attention due to their important roles in many diseases, but the functions and mechanisms of many lncRNAs in HPH are not yet clear. According to the interactions of lncRNAs, miRNAs and mRNAs through miRNA recognition elements [4], a lncRNA–miRNA–mRNA ceRNA network was therefore constructed in the present study to explore the mechanism and reveal novel biomarkers of HPH.

In the present study, we established a HPH rat model via 8 h of hypoxia exposure (10% O_2_) per day for 21 days. Successful construction of a rat HPH model was confirmed by detection of RVSP, RVHI and WA. High-throughput sequencing was performed on the lung tissue to investigate mRNA, lncRNA and miRNA expression level changes in HPH rats versus normal rats. The results revealed 31 up-regulated lncRNAs, 29 down-regulated lncRNAs, 15 up-regulated miRNAs, 5 down-regulated miRNAs, 534 up-regulated mRNAs and 161 down-regulated mRNAs. The expression levels of these RNAs were significantly different between the HPH group and the healthy group, indicating the potential use of these ncRNAs in distinguishing HPH from healthy controls.

To further explore the molecular functions and related pathways of the DE RNAs, GO and KEGG pathways were analysed. The immune response, inflammatory response, cellular response to interleukins, cell cycle, IL-17 signalling pathway, Toll-like receptor signalling pathway and leukocyte migration were highly enriched. These results suggested that many immune and inflammatory response pathways participate in HPH [22,23].

Based on the ceRNA regulatory network theory, we predicted the targets of the DE ncRNAs in the miRbase database to identify possible interactions among the screened DE RNAs. A lncRNA–miRNA–mRNA network was then constructed, and it included 7 lncRNAs, 16 miRNAs and 144 mRNAs. Almost none of the DE lncRNAs we screened have been studied, but many other lncRNAs have been confirmed to be important regulators of PH [24]. LncRNAs TUG1 and NONRATT015587.2, as ceRNAs, have been reported to have an impact on vascular remodelling in PH [25,26]. LncRNA CASC2 and Hoxaas3 regulate PASMC proliferation and migration in HPH by influencing the expression of miR-222 and homeobox a3, respectively [27,28]. LncRNA PAXIP1-AS1 plays an important role in PH by affecting PH-specific transcriptional programmes [29]. These previous results suggest that multiple lncRNAs are involved in HPH through different pathways. Thus, we propose that the seven DE lncRNAs (Ly6l, AABR07038849.2, AABR07069008.2, AABR07064873.1, AABR07001382.1, AABR07068161.1 and AABR07060341.2) in the ceRNA network may serve as molecular sponges of the corresponding miRNAs to regulate mRNA expression and participate in the occurrence and development of HPH by regulating the cell cycle, inorganic molecular entity transmembrane transporter activity, small molecule binding, cytokine–cytokine receptor interaction, p53 signalling pathway and other processes. At present, the role of the cell cycle in the pathogenesis of HPH has been confirmed by many studies [8,30], while the role of other biological processes in HPH has not been studied. Our future research will focus on exploring the roles of DE RNAs in the ceRNA network and the enriched biological processes in HPH pathogenesis.

The functions of some DE miRNAs have been explored. MiR-136-5p has been confirmed to regulate the inflammatory response and is up-regulated in many inflammation-related diseases [31,32]. Joshi et al. found that miR-21-5p is up-regulated in a pulmonary arterial hypertension rat model using an unbiased quantitative miRNA microarray analysis [16], which was consistent with our results. MiR-210-5p promotes epithelial–mesenchymal transformation, an important contributor to the development of HPH, by inhibiting the expression of PIK3R5 [33]. Yan et al. reported that miR-23a is highly expressed in hypoxia-treated pulmonary artery smooth muscle cells (PASMCs) and plays an important role in hypoxia-induced phenotypic transformation of PASMCs [34]. In the past 5 years, miR-23a has been found to be a biomarker for idiopathic PH [35] and a possible therapeutic target for pulmonary arterial hypertension because it promotes cell proliferation and migration by targeting the BMPR2/Smad1 signal in hypoxia-induced PASMCs [36].

In addition to these DE miRNAs, many others have been shown to be involved in the formation of HPH, especially hub genes. Nusap1 regulates the Wnt/β-catenin and TGF-β signalling pathways [37,38], which have been repeatedly reported to be related to HPH pathogenesis [39,40]. Ccnb1 has been confirmed to affect the cell cycle [41], which was indicated in our functional enrichment analysis. Cxcl11 is associated with various inflammatory responses [42,43] and is a critical contributor to HPH.

In general, our study showed differences in the expression levels of lncRNAs, miRNAs and mRNAs between HPH and healthy rats. By constructing a lncRNA–miRNA–mRNA ceRNA network, we identified a regulatory relationship between ncRNAs and mRNAs in addition to their roles in HPH development. Our study contributes to a better understanding of the pathogenesis of HPH and the role of ncRNAs in disease progression.

Although the role of lncRNAs and miRNAs in HPH was successfully analysed and discussed, the present study also had several limitations. First, the sample size was too small (3 HPH rats and 3 healthy rats) to draw conclusions. Second, we only performed qPCR verification, and the results of functional analysis were not experimentally verified. Further research is needed to determine the molecular mechanisms by which ncRNAs play a role in HPH generation and development.

## Supplementary Material

Supplementary Tables S1-S2

## Data Availability

All data has been uploaded to GSE159668. The data sets used and/or analyzed in this study are available from the corresponding author on reasonable request.

## Abbreviations

DE: differentially expressed
GO: Gene Ontology
HPH: hypoxic pulmonary hypertension
KEGG: Kyoto Encyclopaedia of Genes and Genomes
lncRNA: long noncoding RNA
MRE: miRNA response element
PPI: protein–protein interaction
