# Supplementary material for: Construction and analysis of the abnormal lncRNA–miRNA–mRNA network in hypoxic pulmonary hypertension
Source: Biosci Rep. 2021 Aug 26;41(8):BSR20210021. doi: 10.1042/BSR20210021 (PMC8390787; doi:10.1042/BSR20210021)
Supplement: Supplementary Tables S1-S2 [file BSR-2021-0021_supp.zip › BSR-2021-0021_suppST1.pdf]

Supplementary Table 1. Differentially expressed RNAs

Gene name	Category	Log_2FC	P-value
MSTRG.2562.4	lncRNA	-∞	6.92E-10
Cd93	lncRNA	-∞	2.39E-06
MSTRG.12030.2	lncRNA	-∞	7.33E-05
MSTRG.25210.2	lncRNA	-∞	0.000781
MSTRG.10651.2	lncRNA	-∞	0.002835
MSTRG.25922.3	lncRNA	-∞	0.004875
AABR07027581.1	lncRNA	-∞	0.012425
MSTRG.17155.2	lncRNA	-∞	0.012544
MSTRG.14304.2	lncRNA	-∞	0.023616
MSTRG.4797.15	lncRNA	-∞	0.027825
MSTRG.24843.2	lncRNA	-∞	0.029932
AABR07072184.2	lncRNA	-∞	0.033002
MSTRG.25065.4	lncRNA	-∞	0.033076
MSTRG.2562.2	lncRNA	∞	1.81E-08
MSTRG.13187.2	lncRNA	∞	1.84E-06
MSTRG.10462.2	lncRNA	∞	0.000218
MSTRG.3276.2	lncRNA	∞	0.00139
Smg1	lncRNA	∞	0.001416
MSTRG.254.4	lncRNA	∞	0.0052
MSTRG.2562.3	lncRNA	∞	0.007361
MSTRG.12792.5	lncRNA	∞	0.012608
MSTRG.16358.1	lncRNA	∞	0.012659
MSTRG.8583.3	lncRNA	∞	0.02048
Itgb5	lncRNA	∞	0.024884
MSTRG.7257.2	lncRNA	∞	0.027161
MSTRG.10833.24	lncRNA	∞	0.033155
MSTRG.7742.2	lncRNA	∞	0.033913

MSTRG.25065.6	lncRNA	∞	0.034894
MSTRG.10930.2	lncRNA	∞	0.036698
MSTRG.6873.3	lncRNA	∞	0.038681
MSTRG.60.2	lncRNA	∞	0.042148
Astn1	lncRNA	∞	0.044339
MSTRG.14653.9	lncRNA	∞	0.048265
MSTRG.1423.3	lncRNA	13.0138286	1.15E-05
MSTRG.25065.1	lncRNA	9.030167627	0.000295
MSTRG.2386.29	lncRNA	7.576233961	1.88E-05
AABR07051646.1	lncRNA	4.525568596	0.033262
MSTRG.18718.1	lncRNA	4.300216989	0.028546
MSTRG.18548.1	lncRNA	3.827688385	0.010444
MSTRG.6836.1	lncRNA	3.751893307	0.049695
MSTRG.13420.2	lncRNA	2.563315279	0.033109
AABR07060341.2	lncRNA	2.515361608	0.017837
AABR07068161.1	lncRNA	2.511369572	0.007178
MSTRG.21457.1	lncRNA	1.945314694	0.014887
MSTRG.10462.3	lncRNA	-1.847945797	0.031747
AABR07072184.3	lncRNA	-1.986853759	0.04524
MSTRG.1354.1	lncRNA	-2.008863738	0.033601
MSTRG.1423.1	lncRNA	-2.025922751	0.009813
MSTRG.13508.1	lncRNA	-2.07479865	0.017058
AABR07001382.1	lncRNA	-2.252769509	0.006687
Ly6l	lncRNA	-2.262044275	0.025966
AABR07069008.2	lncRNA	-2.747443047	0.033407
MSTRG.333.1	lncRNA	-2.923690064	0.001003
MSTRG.13187.1	lncRNA	-3.665847575	0.000334
MSTRG.254.8	lncRNA	-4.606420266	0.007299
MSTRG.4797.14	lncRNA	-4.986789598	0.007855

MSTRG.17676.1	lncRNA	-5.610949745	0.048885
AABR07064873.1	lncRNA	-5.611997905	2.06E-08
MSTRG.14140.4	lncRNA	-7.394101321	0.000203
AABR07038849.2	lncRNA	-8.554926902	0.001177
rno-miR-1-3p	miRNA	-1.486195591	0.024096
rno-miR-23a-5p	miRNA	-1.365840657	0.021024
rno-miR-293-5p	miRNA	-2.906345113	0.003355
rno-miR-3552	miRNA	-4.341832488	0.028001
rno-miR-483-3p	miRNA	-2.296233469	0.04811
rno-miR-210-3p	miRNA	1.44582087	1.07E-09
rno-miR-21-5p	miRNA	1.339401393	5.25E-07
rno-miR-155-3p	miRNA	2.945122391	8.03E-05
rno-miR-21-3p	miRNA	1.426520846	0.000627
rno-miR-466c-5p	miRNA	1.34151197	0.000833
rno-miR-1193-3p	miRNA	1.039985982	0.0297
rno-miR-136-5p	miRNA	1.086522823	0.017369
rno-miR-210-5p	miRNA	1.359497159	0.005441
rno-miR-223-5p	miRNA	1.110664461	0.001924
rno-miR-299b-3p	miRNA	4.079590258	0.049247
rno-miR-409a-5p	miRNA	1.365209787	0.004594
rno-miR-485-5p	miRNA	1.819833888	0.032588
rno-miR-496-3p	miRNA	1.866976434	0.033663
rno-miR-499-3p	miRNA	2.135574359	0.022686
rno-miR-541-3p	miRNA	2.75698423	0.045737
Reg3g	mRNA	9.243529111	1.02E-05
AABR07030793.1	mRNA	8.625484052	2.91E-06
LOC100909671	mRNA	7.31491464	0.000126
AABR07030143.1	mRNA	7.215993078	0.000392
Slc13a3	mRNA	6.542543434	0.008081

Lefty2	mRNA	6.279047347	3.94E-05
Izumo1r	mRNA	6.249711931	0.000217
Glycam1	mRNA	6.107855085	0.003658
AABR07067882.1	mRNA	6.002271479	0.007729
Tmem213	mRNA	5.852126607	0.009874
AABR07044383.1	mRNA	5.787984415	0.022036
Orm1	mRNA	5.706417713	7.29E-05
Wfdc21	mRNA	5.540832911	0.00013
A2m	mRNA	5.5327688	0.01347
Aqp12a	mRNA	5.495641414	0.000303
AC108576.1	mRNA	5.459073589	0.006223
AC107096.1	mRNA	5.378059307	0.011725
Csf3	mRNA	5.279087553	0.019488
Pla2g2a	mRNA	5.21567267	1.9E-06
St6galnac1	mRNA	5.066991004	0.004679
Tmem235	mRNA	5.015608481	0.003364
Nudt17	mRNA	5.002174004	0.004011
Hoxa1	mRNA	4.863510598	0.021375
AABR07051718.1	mRNA	4.850283815	0.02778
Cxcl6	mRNA	4.776107822	7.51E-05
Defa5	mRNA	4.767131091	0.000168
AABR07034736.1	mRNA	4.623025771	1.2E-08
Chrm1	mRNA	4.610688402	0.012186
Zcchc13	mRNA	4.608530803	0.01211
Catsperz	mRNA	4.592276194	0.018193
Trim29	mRNA	4.55926414	0.003924
Slc7a9	mRNA	4.554158578	0.000138
Scn4b	mRNA	4.527633993	0.003189
AABR07051733.1	mRNA	4.480528903	5.89E-05

Adamts16	mRNA	4.457879545	0.023729
AABR07008370.1	mRNA	4.457029386	0.044236
AABR07017250.1	mRNA	4.444459267	0.037923
Np4	mRNA	4.427403482	7.03E-09
Itln1	mRNA	4.421123039	0.000382
Mpo	mRNA	4.376952482	1.04E-05
AC094246.1	mRNA	4.272023216	0.033396
Best2	mRNA	4.253566545	0.037956
AABR07040288.1	mRNA	4.145883819	0.00019
Wfdc15b	mRNA	4.101799505	1.77E-07
AABR07051556.1	mRNA	3.995073609	0.000121
AABR07013111.1	mRNA	3.966329116	0.000194
Reg3b	mRNA	3.919884452	0.003718
AABR07051548.2	mRNA	3.906304485	0.001132
Aldh1l2	mRNA	3.843202472	0.001828
AABR07051708.1	mRNA	3.834030236	2.24E-05
AABR07051733.2	mRNA	3.792882575	1.47E-05
Calcb	mRNA	3.77783484	0.012906
Sult1b1	mRNA	3.726485007	0.007082
Timp1	mRNA	3.724837037	0.000122
AABR07006033.1	mRNA	3.724570757	0.003628
Mmp7	mRNA	3.723223324	0.010172
Ifitm6	mRNA	3.66598359	5.75E-18
AABR07065792.2	mRNA	3.645095117	0.00021
AABR07051551.1	mRNA	3.637133274	0.000122
AABR07065837.1	mRNA	3.608472015	5.95E-07
Cpxm1	mRNA	3.532728339	6.09E-05
Cxcl13	mRNA	3.521579914	2E-05
Bpifb1	mRNA	3.44037462	0.001294

Pla2g4c	mRNA	3.428896138	0.001366
Brs3	mRNA	3.425975242	0.001869
Spin2a	mRNA	3.416454093	0.007465
Tmem132d	mRNA	3.37256311	0.00048
Cd177	mRNA	3.318331245	8.62E-06
Il19	mRNA	3.29774064	0.029044
Ankrd34a	mRNA	3.296155499	0.000451
Cxcl9	mRNA	3.254608059	0.001016
Bpifb2	mRNA	3.246756786	0.019927
Ngp	mRNA	3.245865688	6.91E-17
RGD1563231	mRNA	3.183291426	2.68E-06
S100a8	mRNA	3.177458818	1.03E-11
AABR07065821.1	mRNA	3.166951654	0.000101
AABR07061136.1	mRNA	3.166725192	0.011375
Ms4a12	mRNA	3.123710758	0.008229
Prss29	mRNA	3.108937804	0.000712
AABR07051684.1	mRNA	3.107164804	0.000146
Mmp9	mRNA	3.091385883	4.6E-13
RGD1310495	mRNA	3.088939316	0.020817
AABR07013843.1	mRNA	3.071019692	0.005574
Wt1	mRNA	3.057620755	0.005104
AABR07060980.1	mRNA	3.048564214	9.66E-05
AABR07051551.2	mRNA	3.021697459	1.64E-06
Slc32a1	mRNA	3.01226426	0.015111
Fcrl2	mRNA	3.001878625	0.002621
AABR07065699.4	mRNA	2.990732685	0.014673
AABR07065693.3	mRNA	2.983855018	8.37E-05
AABR07051746.1	mRNA	2.969084205	0.001775
Cxcl11	mRNA	2.965390339	0.007318

AABR07051565.1	mRNA	2.92141358	0.013757
AABR07056026.1	mRNA	2.920918999	8.9E-06
AABR07051658.1	mRNA	2.903103529	0.018735
Prss30	mRNA	2.900887947	0.000653
AABR07006032.1	mRNA	2.895868772	0.045617
AABR07051626.2	mRNA	2.893203189	0.03767
Ptprv	mRNA	2.876895089	0.000696
Prss34	mRNA	2.862574964	0.034156
Gpr33	mRNA	2.859737202	0.013696
AC107446.2	mRNA	2.859217594	0.035179
Sdr16c5	mRNA	2.849127265	0.035289
AABR07061001.1	mRNA	2.819619133	2.54E-06
S100a9	mRNA	2.802934908	1.79E-11
AABR07051563.1	mRNA	2.774998494	0.000768
AABR07034730.3	mRNA	2.766639651	0.000116
Pycr1	mRNA	2.765035164	0.00901
Shisa3	mRNA	2.764075219	0.034169
LOC100909700	mRNA	2.757466303	0.000104
AABR07060963.1	mRNA	2.722993115	0.001132
Nuggc	mRNA	2.717936448	0.000655
AABR07065886.1	mRNA	2.703990161	0.004587
AABR07065778.3	mRNA	2.703263791	1.74E-06
Cplx4	mRNA	2.702285557	0.015172
Lmo1	mRNA	2.692736775	0.013677
Ccl7	mRNA	2.683450027	0.038404
AABR07060952.1	mRNA	2.683413083	0.001834
Slurp1	mRNA	2.683059633	0.047516
Igh-6	mRNA	2.66954118	7.2E-08
Ctsg	mRNA	2.655032054	0.010102

Prokr2	mRNA	2.636829042	0.002691
Aldh18a1	mRNA	2.630985515	0.036928
AABR07065813.1	mRNA	2.629022809	0.033127
Fstl4	mRNA	2.624787247	0.001936
AABR07065792.1	mRNA	2.61019535	0.001123
AABR07072262.1	mRNA	2.606747455	0.011818
Elovl2	mRNA	2.596456499	0.004903
AABR07051583.1	mRNA	2.580275822	0.001112
AABR07065750.2	mRNA	2.561013886	8.6E-07
AC109901.2	mRNA	2.558920185	3.32E-05
P2rx5	mRNA	2.544268127	0.005833
AABR07034739.2	mRNA	2.543403328	0.00315
Atp6v0a4	mRNA	2.535062742	0.002537
Camp	mRNA	2.532965238	1.24E-06
AABR07065798.1	mRNA	2.53114737	0.016375
AABR07065772.2	mRNA	2.524738726	0.000451
Mmp8	mRNA	2.502310254	5.34E-07
Gp2	mRNA	2.501340705	0.008986
Lcn2	mRNA	2.500992075	0.000974
Npy4r	mRNA	2.483503201	0.000176
Scgb3a1	mRNA	2.468023589	0.009211
Slpi	mRNA	2.462168703	1.8E-07
AABR07061178.1	mRNA	2.444271016	0.006053
Dmrt2	mRNA	2.443253186	0.033094
Klra5	mRNA	2.435371348	0.006284
AABR07034739.1	mRNA	2.433152315	3.23E-08
Ces2g	mRNA	2.420627629	0.037446
Sap18	mRNA	2.396982154	0.015635
Krt81	mRNA	2.387382685	0.003906

Pla2g5	mRNA	2.384022707	0.02144
AABR07065789.1	mRNA	2.380094679	0.002583
Rspo3	mRNA	2.367430285	0.02404
Mgam	mRNA	2.356326673	2.56E-09
AABR07034730.2	mRNA	2.346861539	6.93E-05
AABR07020815.1	mRNA	2.341666338	0.007905
Ptprn	mRNA	2.316591816	0.00229
Prg2	mRNA	2.31103619	0.000608
AABR07007853.1	mRNA	2.309893909	0.043816
LOC685048	mRNA	2.308320667	0.002375
Plin1	mRNA	2.300396789	0.044647
C6	mRNA	2.293633895	7.63E-08
Spp1	mRNA	2.292036262	6.01E-05
AABR07065823.2	mRNA	2.278712107	0.002972
Traip	mRNA	2.278519627	0.001691
Ntsr1	mRNA	2.276030886	0.011607
AABR07065823.1	mRNA	2.271292031	0.001525
Prok2	mRNA	2.256127135	3.24E-05
Rbp2	mRNA	2.250954694	0.047663
AABR07051670.1	mRNA	2.244589645	0.006105
LOC292543	mRNA	2.240321136	0.001473
AABR07065778.2	mRNA	2.233902586	0.010396
Thbs2	mRNA	2.227933774	0.000795
AABR07065883.1	mRNA	2.224693613	4.95E-05
Neil3	mRNA	2.218077171	0.008459
Aicda	mRNA	2.217863343	0.010786
Knstrn	mRNA	2.21352691	0.002481
Ccl19	mRNA	2.183084034	0.000419
Serpinb11	mRNA	2.177945498	0.029986

AABR07051733.3	mRNA	2.176037215	0.006469
AABR07029275.1	mRNA	2.172390874	0.003049
Epb41l1	mRNA	2.167591031	0.035681
Cyp26a1	mRNA	2.166355523	0.02199
LOC100910446	mRNA	2.160266083	0.020383
Abca13	mRNA	2.151420107	0.000499
AABR07034729.1	mRNA	2.142785521	0.02428
Serpinb2	mRNA	2.12595338	0.039871
Clec4b2	mRNA	2.107268861	0.000111
Steap1	mRNA	2.095503265	0.005698
Muc5b	mRNA	2.088669445	0.002972
Fpr1	mRNA	2.078406582	0.000133
Xrcc3	mRNA	2.075696599	0.039841
AABR07065789.3	mRNA	2.059567829	0.000599
Arhgef39	mRNA	2.057622425	0.007757
AABR07065823.3	mRNA	2.051262536	0.001419
Rbp7	mRNA	2.049800094	0.023729
Exo1	mRNA	2.049103245	0.001134
Mzb1	mRNA	2.02500784	2.08E-05
Lilrb3	mRNA	2.024452629	0.006897
Sgo1	mRNA	2.023731033	0.001179
Areg	mRNA	2.019588739	5.19E-06
Pdcd1	mRNA	2.015202928	0.001317
AABR07051532.1	mRNA	2.005632117	0.002346
Golga7b	mRNA	2.004621647	0.022821
Xkr5	mRNA	2.002854762	0.024411
Mfrp	mRNA	2.00188058	0.037818
Ildr2	mRNA	1.995328398	0.040006
AABR07060872.1	mRNA	1.995247066	1.29E-05

AABR07065815.2	mRNA	1.985112493	0.049694
Ptges	mRNA	1.972895878	0.038463
Crhr2	mRNA	1.951943107	0.012764
Kif2c	mRNA	1.945442425	0.004411
Serp∞1	mRNA	1.940432108	0.001629
Capn12	mRNA	1.939515673	0.009108
AABR07065814.2	mRNA	1.932673827	0.004929
Fam110c	mRNA	1.929403874	0.002765
C1s	mRNA	1.92615374	3.17E-05
Lypd6b	mRNA	1.924223509	0.046418
Hs6st2	mRNA	1.920487213	0.016922
Ccne1	mRNA	1.911988077	0.002119
LOC689230	mRNA	1.908695517	0.035322
Shcbp1	mRNA	1.904506168	0.003889
Asf1b	mRNA	1.901854016	0.002221
Cenpw	mRNA	1.896298685	0.01672
Dhrs9	mRNA	1.894656829	0.003315
Dctd	mRNA	1.887138072	3.28E-05
Pbk	mRNA	1.884198449	0.003075
AABR07065781.1	mRNA	1.878314787	0.00249
Kansl2	mRNA	1.85564714	0.04822
AABR07065834.1	mRNA	1.852022842	0.022755
AABR07002848.1	mRNA	1.850702465	0.036676
Ccl2	mRNA	1.847131192	0.015531
Oip5	mRNA	1.836955717	0.021931
RGD1560925	mRNA	1.833151121	0.021171
Jchain	mRNA	1.831089091	0.000101
Tfpi2	mRNA	1.828599781	0.017281
Kif20a	mRNA	1.824910434	0.003976

Ubd	mRNA	1.82086878	0.022047
Cd300c2	mRNA	1.816832761	0.000854
Cacng6	mRNA	1.81513482	0.011589
Retnlg	mRNA	1.808855224	2.05E-11
Cenpu	mRNA	1.808682928	0.011656
Serpinb1a	mRNA	1.805356979	8.1E-19
Birc5	mRNA	1.803448938	0.004604
Ibsp	mRNA	1.799044556	0.00506
Dsg3	mRNA	1.796957597	0.019851
Folr2	mRNA	1.792365214	0.004154
LOC103694908	mRNA	1.79227416	0.006451
Cdca3	mRNA	1.778918819	0.009972
Ttk	mRNA	1.774522752	0.003929
Cebpe	mRNA	1.772397465	0.004718
Adgrg2	mRNA	1.759410215	0.037667
Ccnb1	mRNA	1.757198836	0.004173
Ankle1	mRNA	1.754495136	0.016977
Ncapg	mRNA	1.748910243	0.001467
Crmp1	mRNA	1.747870989	0.001019
F2rl2	mRNA	1.747386379	8.56E-07
AC128848.1	mRNA	1.745117453	0.001287
Ccl11	mRNA	1.744775725	1.41E-09
Msln	mRNA	1.744556348	0.023852
Rgsl1	mRNA	1.743900955	0.001761
Pnpla1	mRNA	1.742047423	0.041713
Dtl	mRNA	1.741151648	0.001
Slc36a2	mRNA	1.739853936	0.037957
Hist1h2ao	mRNA	1.734873657	0.006144
Cep55	mRNA	1.733064716	0.00613

Ect2	mRNA	1.731602827	0.00213
Il1r2	mRNA	1.726949444	0.001124
AABR07051562.1	mRNA	1.717385098	0.045468
AABR07027447.1	mRNA	1.714371035	0.048579
Rad51	mRNA	1.713888622	0.007452
Sapcd2	mRNA	1.712527916	0.010648
Mastl	mRNA	1.705195952	0.004088
Hist1h3b	mRNA	1.704286281	0.002035
Acod1	mRNA	1.702829589	0.035162
Pclaf	mRNA	1.68760856	0.016571
Ano2	mRNA	1.687009027	0.005863
Tk1	mRNA	1.686474986	0.002482
Bub1b	mRNA	1.685948155	0.004494
Cxcr2	mRNA	1.679383309	3.99E-07
Mt2A	mRNA	1.67785408	0.000114
Top2a	mRNA	1.667228805	0.000675
LOC100911417	mRNA	1.662276682	0.016456
Cdkn3	mRNA	1.659903397	0.013873
Rrm2	mRNA	1.654815245	0.000496
RGD1307182	mRNA	1.654350575	0.003592
Ccna2	mRNA	1.649875828	0.002879
Ube2c	mRNA	1.648734728	0.020864
Tmco2	mRNA	1.636242528	0.010402
Dctpp1	mRNA	1.634576333	0.027519
Hist1h1a	mRNA	1.633363326	0.000863
AABR07027451.1	mRNA	1.627362492	0.011674
Kifc1	mRNA	1.62536105	0.008568
Ube2t	mRNA	1.622048185	0.02146
Bard1	mRNA	1.61748561	0.002292

Slc38a11	mRNA	1.61598709	0.027956
LOC689757	mRNA	1.611449839	0.031112
Hist1h2ai	mRNA	1.61009996	0.001705
Nusap1	mRNA	1.606485063	0.005872
Nuf2	mRNA	1.60333506	0.003182
LOC498276	mRNA	1.600685522	4.8E-09
Spc24	mRNA	1.591448818	0.023178
LOC100909595	mRNA	1.589914818	8.55E-10
Mt1	mRNA	1.585679905	0.000157
Clec4e	mRNA	1.582426893	0.000335
Tnfrsf17	mRNA	1.581603394	0.008283
Melk	mRNA	1.580655325	0.011636
Troap	mRNA	1.578905928	0.042527
Trh	mRNA	1.571228579	0.032933
AABR07061005.2	mRNA	1.570865008	0.016484
AABR07028352.1	mRNA	1.57041436	0.048501
LOC100359539	mRNA	1.56085518	0.000775
Kif22	mRNA	1.556109852	0.006365
Ltb4r	mRNA	1.555455793	0.04368
Ramp3	mRNA	1.553064224	9.32E-07
Tpx2	mRNA	1.544478131	0.00439
Selp	mRNA	1.543983237	0.001023
LOC103690190	mRNA	1.541977708	0.001822
Baat	mRNA	1.539006081	0.000137
Ncaph	mRNA	1.534360069	0.002491
Ms4a7	mRNA	1.533660577	0.013983
Cenpe	mRNA	1.530764862	0.001201
Spag5	mRNA	1.512981056	0.008567
Hjurp	mRNA	1.510586775	0.007825

AABR07065625.1	mRNA	1.509779841	9.71E-06
LOC100910554	mRNA	1.508403814	0.011774
E2f8	mRNA	1.507054147	0.017956
Cfd	mRNA	1.504790488	0.000459
Cenpk	mRNA	1.503314717	0.009265
Ccdc80	mRNA	1.502540272	0.002283
Hist1h2bh	mRNA	1.49784426	0.013471
Ctla4	mRNA	1.489937075	0.000449
Gfpt2	mRNA	1.489642683	0.008187
Nek2l1	mRNA	1.482586727	0.005336
AABR07060886.1	mRNA	1.48244826	0.001146
Ecel1	mRNA	1.473608932	0.018741
Col1a1	mRNA	1.472574569	0.027844
Ticrr	mRNA	1.472235874	0.006847
Cenpt	mRNA	1.472021809	0.033282
Kif20b	mRNA	1.469676285	0.005726
Mki67	mRNA	1.466514288	0.001456
Tarm1	mRNA	1.462262109	0.015145
Cenph	mRNA	1.459929671	0.02539
Dsg1	mRNA	1.456248667	0.005824
Gas2l3	mRNA	1.455251018	0.012283
Ism2	mRNA	1.453316311	0.016719
Apobec1	mRNA	1.434004608	0.00368
Ptn	mRNA	1.431488751	0.002845
Cdca8	mRNA	1.430626576	0.005773
AABR07063346.1	mRNA	1.424540376	0.049451
Gpr39	mRNA	1.42047943	0.030895
Trem2	mRNA	1.417658447	0.021974
Mcm10	mRNA	1.414907468	0.003195

Ddias	mRNA	1.413880431	0.040582
Rarres1	mRNA	1.40548289	0.004117
Orc1	mRNA	1.404938295	0.004121
Ska1	mRNA	1.402121507	0.046351
Ckap2l	mRNA	1.401623624	4.19E-05
Hmmr	mRNA	1.396961889	0.006823
Tifab	mRNA	1.393532398	0.007572
Hist1h2ah	mRNA	1.392860277	0.001587
Mybl2	mRNA	1.392645852	0.022188
Padi4	mRNA	1.390790935	7.12E-05
Cd300le	mRNA	1.389736039	0.000784
AABR07032097.1	mRNA	1.388927567	0.032245
AABR07003235.1	mRNA	1.388346189	0.01159
Sgo2	mRNA	1.382826566	0.00843
Krt78	mRNA	1.377450792	0.023373
Bcat1	mRNA	1.375460229	0.002836
Slc2a5	mRNA	1.372690786	0.026468
Chrd	mRNA	1.372393629	0.003839
Cma1	mRNA	1.372244946	0.014566
Pttg1	mRNA	1.368940351	0.029352
Cks2	mRNA	1.366291751	0.015443
Rps10l1	mRNA	1.365981509	3.73E-05
AABR07066818.1	mRNA	1.363886949	0.003036
Uhrf1	mRNA	1.363472184	0.0049
Pkhd1l1	mRNA	1.362071937	0.016579
Hk3	mRNA	1.356954634	2.47E-05
Kif18b	mRNA	1.354391127	0.023765
Nos2	mRNA	1.354223896	0.017231
Kntc1	mRNA	1.35094219	0.009062

Cenpf	mRNA	1.347168028	0.010809
Hist1h3a	mRNA	1.343192585	0.006698
Emilin2	mRNA	1.341660107	6.28E-08
Smpd3	mRNA	1.340692405	0.003721
Hist1h2ail1	mRNA	1.340012981	0.014765
Tpsab1	mRNA	1.336165346	0.040359
Penk	mRNA	1.334759446	0.020195
Steap4	mRNA	1.333880237	0.000952
Gins1	mRNA	1.326739453	0.014774
Cdc6	mRNA	1.325650996	0.011414
Spc25	mRNA	1.31651357	0.037006
Pram1	mRNA	1.315200545	0.003293
Mefv	mRNA	1.314119358	4.29E-07
Sphk1	mRNA	1.314064757	0.001798
Mcpt1l1	mRNA	1.312726231	0.005025
LOC684773	mRNA	1.305072421	0.004014
LOC103694905	mRNA	1.303133278	0.040393
Entpd2	mRNA	1.302795383	0.005357
Csf3r	mRNA	1.302397186	0.000172
Adgrd1	mRNA	1.301215345	0.038543
Kif11	mRNA	1.29982708	0.006657
Itgad	mRNA	1.299527757	1.96E-05
Anln	mRNA	1.295684432	0.011969
Il12a	mRNA	1.29377821	0.00686
Pla2g7	mRNA	1.293633696	0.000611
Mms22l	mRNA	1.28924432	0.006538
Nlrp12	mRNA	1.28601652	0.002632
Cenpa	mRNA	1.283735161	0.042728
Tgfbi	mRNA	1.278238956	4.87E-05

Depdc1	mRNA	1.278125122	0.019704
Rps6	mRNA	1.268783348	0.042662
Pdgfc	mRNA	1.268222507	0.000408
Dlgap5	mRNA	1.268095749	0.009012
Tnfrsf9	mRNA	1.267482835	0.011194
Slc9a2	mRNA	1.267134749	0.0412
Cadm4	mRNA	1.267131293	0.011381
Cdk1	mRNA	1.264610216	0.023847
Mreg	mRNA	1.261915993	0.003323
Fndc1	mRNA	1.257590838	0.048688
AABR07027809.1	mRNA	1.251215007	0.038971
Vcan	mRNA	1.24915104	0.002971
Fpr2	mRNA	1.247419716	0.008237
AABR07051611.1	mRNA	1.246448415	0.042582
Clec10a	mRNA	1.245454385	0.000134
P2ry6	mRNA	1.238803554	0.010753
Hist1h2an	mRNA	1.236501691	0.001072
Diaph3	mRNA	1.232851325	0.012416
Hist1h2ak	mRNA	1.232065356	0.004721
Kif14	mRNA	1.229201981	0.027113
Mcm5	mRNA	1.227659119	0.004098
C3	mRNA	1.226153006	0.001857
Cenpi	mRNA	1.220606986	0.022724
Hist1h2bk	mRNA	1.219299178	0.0094
Tigit	mRNA	1.218460049	0.03537
Slamf9	mRNA	1.216132982	0.042674
Cdc20	mRNA	1.214451523	0.029259
LOC100911825	mRNA	1.213411912	0.01152
Ereg	mRNA	1.213138493	0.01554

Hist1h1b	mRNA	1.209118247	0.001259
Tubg2	mRNA	1.20626521	0.033005
Csf2rb	mRNA	1.205973763	8.95E-07
C4b	mRNA	1.198960713	0.000162
Tsku	mRNA	1.191011413	0.005058
LOC100134871	mRNA	1.186324792	0.000158
Scimp	mRNA	1.185592215	1.6E-05
Esco2	mRNA	1.185226911	0.013192
Col5a3	mRNA	1.183275504	0.048789
C1qb	mRNA	1.183206528	0.000916
LOC102548682	mRNA	1.181403109	0.043306
Fcgr2b	mRNA	1.181154601	0.000216
Lgals1	mRNA	1.180798776	0.010364
Fanci	mRNA	1.179123195	0.016751
Racgap1	mRNA	1.174543773	0.012634
AABR07071838.1	mRNA	1.166718759	0.008685
Oscar	mRNA	1.16148612	0.027245
Lrg1	mRNA	1.159127333	8.45E-07
Hmox1	mRNA	1.155399093	0.015756
Stil	mRNA	1.152655651	0.013999
Asgr2	mRNA	1.150775152	0.030372
Rufy4	mRNA	1.150391345	0.00724
Slc17a2	mRNA	1.145929571	0.028147
LOC691143	mRNA	1.140404275	0.012741
Fanca	mRNA	1.136952121	0.02816
Hist1h2bl	mRNA	1.135563903	0.048552
Iqgap3	mRNA	1.135478295	0.028825
Gpt2	mRNA	1.134384549	0.005679
Esrrb	mRNA	1.133840688	0.030518

Kcng4	mRNA	1.130329209	0.035849
Cyp4f18	mRNA	1.129156864	0.000414
Nfil3	mRNA	1.128499231	0.024461
Aspm	mRNA	1.127497513	0.005622
Chi3l1	mRNA	1.127171132	1.1E-06
Mcm6	mRNA	1.121406129	0.002286
Tfec	mRNA	1.119531274	0.009002
LOC100911204	mRNA	1.116657279	0.043598
Cyp2e1	mRNA	1.115898093	0.002352
Egr1	mRNA	1.115872993	0.000512
Ptafr	mRNA	1.115385911	8.83E-05
Fancd2	mRNA	1.111384489	0.008451
Cercam	mRNA	1.110030122	0.033465
Gpc1	mRNA	1.106590119	0.000916
Tnnc1	mRNA	1.10579542	0.017381
Parpbp	mRNA	1.099411434	0.048795
Slc7a11	mRNA	1.09897969	0.003437
Clec4a1	mRNA	1.098014935	0.005611
AABR07051700.1	mRNA	1.09568537	0.039604
Pglyrp1	mRNA	1.092764159	0.00015
Haus4	mRNA	1.090710218	0.007851
Glrx	mRNA	1.081720703	0.000115
Ckap2	mRNA	1.080760809	0.035472
Ms4a6a	mRNA	1.080477173	0.010504
Trim72	mRNA	1.078989651	0.020167
Cyp39a1	mRNA	1.078277814	0.014036
C1qtnf5	mRNA	1.07228478	0.000987
AABR07012795.1	mRNA	1.070337838	0.048527
Fos	mRNA	1.069461147	0.000443

MGC105649	mRNA	1.067735925	0.000272
Ndc80	mRNA	1.067374008	0.033393
Susd3	mRNA	1.064307123	0.002711
Clec4a3	mRNA	1.063745594	0.013912
Irf4	mRNA	1.063699591	0.000865
Adgre1	mRNA	1.062444961	0.014307
Cd33	mRNA	1.056451516	0.009002
LOC684762	mRNA	1.056208452	0.005979
Glt8d2	mRNA	1.056190897	0.005809
Polq	mRNA	1.046789229	0.017661
Pstpip1	mRNA	1.045538055	0.000318
Knl1	mRNA	1.044569339	0.017804
Plk1	mRNA	1.041971897	0.038731
C1qa	mRNA	1.041439033	0.010705
P2ry13	mRNA	1.036035461	0.005199
Hist1h2bcl1	mRNA	1.034632553	0.031088
Cip2a	mRNA	1.034255787	0.040339
Rcn3	mRNA	1.034216256	0.011356
Cst7	mRNA	1.033378765	0.00742
Ptger2	mRNA	1.0333176	0.009431
Prrx1	mRNA	1.031614881	0.034709
Bub1	mRNA	1.028277649	0.023422
Rad54b	mRNA	1.02801057	0.032769
Ska3	mRNA	1.027680325	0.048023
Cotl1	mRNA	1.025554532	0.003274
Cdca2	mRNA	1.020775509	0.030203
Eqtn	mRNA	1.013612056	0.046088
Nrn1	mRNA	1.012853717	0.000333
Tlr2	mRNA	1.010864206	3.4E-05

Pkmyt1	mRNA	1.009144237	0.032475
Aif1	mRNA	1.007112277	0.00422
Adora1	mRNA	1.00503998	0.01261
Cd163	mRNA	1.002314105	0.001211
Incenp	mRNA	1.002297668	0.004777
Clec4d	mRNA	1.00142567	0.009261
Ier3	mRNA	1.001175235	0.000587
Cyb5r2	mRNA	-1.000643458	0.008031
Fam151a	mRNA	-1.00315948	0.013889
Wdcp	mRNA	-1.009092042	0.023769
Adcyap1r1	mRNA	-1.016632904	0.034362
Hepacam2	mRNA	-1.026451738	0.003603
Ifit3	mRNA	-1.035248392	0.001002
Apol9a	mRNA	-1.036878341	0.000767
Ano7	mRNA	-1.0370696	0.013951
Cpvl	mRNA	-1.044065386	0.00046
Arfgef3	mRNA	-1.046178335	0.002586
Corin	mRNA	-1.049160257	0.003445
Fbxo39	mRNA	-1.059302098	0.047961
AC120568.1	mRNA	-1.07911521	5.95E-05
Sry	mRNA	-1.092735871	0.001873
Fam129c	mRNA	-1.097844878	0.016986
AABR07066144.1	mRNA	-1.101026955	0.025593
Rtp4	mRNA	-1.106886094	4.33E-07
Sez6l	mRNA	-1.111943767	0.011595
Pou2f3	mRNA	-1.136178195	0.014153
Trpm5	mRNA	-1.139958206	1.6E-05
Tmem229a	mRNA	-1.144499711	0.011462
AABR07025140.1	mRNA	-1.149420544	2.73E-09

Herc6	mRNA	-1.154604439	2.49E-06
Itprid1	mRNA	-1.160043406	0.000171
Asb15	mRNA	-1.174910548	0.010038
Rsad2	mRNA	-1.176599682	1.8E-05
LOC498675	mRNA	-1.187405304	0.012541
Cxcl14	mRNA	-1.199195646	0.001378
Mx1	mRNA	-1.212770411	9.33E-08
Nrap	mRNA	-1.215552976	0.045141
Mycn	mRNA	-1.22271519	0.028583
Gng13	mRNA	-1.243651536	0.016778
Slc28a3	mRNA	-1.24451131	0.001074
RT1-T24-3	mRNA	-1.250010555	1.95E-09
AABR07058464.1	mRNA	-1.252167811	3.23E-06
Myo1a	mRNA	-1.252365978	0.006013
Irf7	mRNA	-1.256912354	4.97E-06
Acsbg1	mRNA	-1.278452292	0.002051
Znf354b	mRNA	-1.285539614	0.002128
Fam229a	mRNA	-1.290836439	0.037884
Lhfpl1	mRNA	-1.294158447	0.007271
AABR07025272.1	mRNA	-1.308690049	4.65E-09
Clec4g	mRNA	-1.323244947	0.019626
Mx2	mRNA	-1.332052691	6.23E-06
Hnf4g	mRNA	-1.33494324	0.006776
Gcnt7	mRNA	-1.338752452	0.028657
Eln	mRNA	-1.351865306	0.000243
Fcer2	mRNA	-1.358425935	0.011325
Lnpep	mRNA	-1.365186688	0.023899
Usp18	mRNA	-1.369728804	5.93E-07
S100g	mRNA	-1.374081644	0.002657

Sh2d7	mRNA	-1.376867602	0.000173
Pkd2l2	mRNA	-1.391483632	0.032965
Tnni3	mRNA	-1.394346827	0.006458
Impg1	mRNA	-1.396331295	0.002034
Rgs17	mRNA	-1.398842046	0.007317
Esrrg	mRNA	-1.414685795	0.035571
AABR07021946.1	mRNA	-1.432207062	0.038393
RGD1562726	mRNA	-1.471021184	0.005955
Spag16	mRNA	-1.472466147	0.034803
Adh7	mRNA	-1.477101459	0.047702
Isg15	mRNA	-1.489126101	4.62E-06
RGD1562914	mRNA	-1.491805922	0.007209
Edar	mRNA	-1.509486047	0.003078
AC094647.2	mRNA	-1.520813135	0.014658
AABR07061801.2	mRNA	-1.532007937	0.035611
Six1	mRNA	-1.536552828	0.01063
AC118957.1	mRNA	-1.558530137	0.04447
Clec18a	mRNA	-1.607605627	0.016141
AABR07037447.1	mRNA	-1.610143542	0.049231
Gbp3	mRNA	-1.615787896	0.000985
Cpne4	mRNA	-1.630927139	0.047827
Gpr165	mRNA	-1.68801766	0.004458
Gnat3	mRNA	-1.699535058	2.45E-05
Nr1i2	mRNA	-1.710613668	0.01203
Syt6	mRNA	-1.729935272	3.82E-06
Oas2	mRNA	-1.745040586	2.19E-06
Nr1d1	mRNA	-1.796748552	0.003906
LOC685767	mRNA	-1.861725823	5.25E-06
Hmx3	mRNA	-1.862239618	0.037823

Efhd1	mRNA	-1.873605809	0.002399
Adamts18	mRNA	-1.899854001	0.037417
Npas1	mRNA	-1.910767172	0.037212
Rnf17	mRNA	-1.934415514	0.000585
AABR07052585.1	mRNA	-1.976536393	0.045184
Npas2	mRNA	-1.999758912	0.00031
Ankrd35	mRNA	-2.01529515	0.007566
Sostdc1	mRNA	-2.034187211	0.029841
Mov10l1	mRNA	-2.052720367	0.011265
RGD1564937	mRNA	-2.056510309	0.041974
LOC688649	mRNA	-2.085731092	0.032178
Qrich2	mRNA	-2.112365139	0.042312
Xirp2	mRNA	-2.118424764	0.001406
Ptcra	mRNA	-2.142116206	0.049031
Pcdh15	mRNA	-2.149907537	0.046195
Spon2	mRNA	-2.21764995	8.05E-10
Nags	mRNA	-2.235393907	0.000134
Zc2hc1b	mRNA	-2.266206813	0.036295
Cpne5	mRNA	-2.317057099	0.005735
Th	mRNA	-2.344110194	0.040233
Kcna6	mRNA	-2.37852233	0.006114
Magee2	mRNA	-2.423841044	0.046244
Fam228a	mRNA	-2.488221208	0.013169
Senp17	mRNA	-2.50647702	0.011781
Inca1	mRNA	-2.512072086	0.011729
AABR07042633.1	mRNA	-2.519066671	0.013558
Cib4	mRNA	-2.536270486	0.039286
AF529169	mRNA	-2.538779712	0.003833
AABR07009260.1	mRNA	-2.549949229	0.032663

Myoc	mRNA	-2.566380084	0.041039
Unc45b	mRNA	-2.651039175	0.004785
Spock1	mRNA	-2.654988071	0.045483
Ltk	mRNA	-2.683907466	0.001358
RGD1560028	mRNA	-2.721555734	0.039339
Tigd4	mRNA	-2.723494972	0.005002
LOC684466	mRNA	-2.75582219	0.01866
Nkx6-1	mRNA	-2.762443382	0.017233
Kcnk16	mRNA	-2.821378841	0.025447
Glipr1l1	mRNA	-2.838863691	0.008769
RT1-Db2	mRNA	-2.977499432	0.000462
Adprhl1	mRNA	-3.07412136	0.031491
LOC500827	mRNA	-3.078865707	0.028331
LOC102552326	mRNA	-3.11017083	0.049573
Acot4	mRNA	-3.118478628	0.035707
Cyp2j16	mRNA	-3.129304727	0.049844
Apoh	mRNA	-3.224327992	0.043
Nppb	mRNA	-3.225685882	0.015069
Akr1c12	mRNA	-3.233815401	0.001402
AABR07034008.1	mRNA	-3.353087695	0.014641
Fmo9	mRNA	-3.375280576	0.029821
Htr5b	mRNA	-3.636625949	0.014126
Asb14	mRNA	-3.944829578	0.001501
AABR07049499.1	mRNA	-4.324731454	0.037518
Naglt1	mRNA	-4.493705741	0.034837
Myl7	mRNA	-4.500550462	0.002147
Iqcf3	mRNA	-4.51268438	0.020373
Olr1398	mRNA	-4.512684571	0.020368
AABR07000738.1	mRNA	-4.520012759	0.022858

Aanat	mRNA	-4.524727991	0.036767
AABR07024769.1	mRNA	-4.53808518	0.037503
Etnppl	mRNA	-4.669212193	0.018113
Krt36	mRNA	-4.670902825	0.025003
Defb25	mRNA	-4.675404758	0.011927
Gfral	mRNA	-4.686001773	0.027438
Fam186b	mRNA	-4.687492478	0.015769
Synpr	mRNA	-4.707833445	0.026423
LOC296235	mRNA	-4.720081392	0.047076
AABR07058519.1	mRNA	-4.723116484	0.036628
Rnase13	mRNA	-4.812407359	0.009389
Olr313	mRNA	-4.826852761	0.007001
Adam3a	mRNA	-4.842014534	0.018264
Gabra5	mRNA	-4.848088913	0.019325
Btnl3	mRNA	-4.972931427	0.01364
Slc6a19	mRNA	-5.290050484	0.001303
Myadml2	mRNA	-5.292043994	0.001009
Gabra6	mRNA	-5.446005237	0.0158
LOC102555392	mRNA	-5.484709317	0.001961
Zdhhc22	mRNA	-5.495754206	0.004395
Capns1	mRNA	-5.710653639	0.022732
Mb	mRNA	-6.841743769	0.000329
Myh6	mRNA	-6.85865462	6.1E-05
